# PKMYT1, exacerbating the progression of clear cell renal cell carcinoma, is implied as a biomarker for the diagnosis and prognosis

**DOI:** 10.18632/aging.203759

**Published:** 2021-12-27

**Authors:** Juan Chen, Xiaoliang Hua, Heying Chen, Xiangmin Qiu, Haibing Xiao, Shengdong Ge, Chaozhao Liang, Qin Zhou

**Affiliations:** 1 The Ministry of Education Key Laboratory of Laboratory Medical Diagnostics, The College of Laboratory Medicine, Chongqing Medical University, Chongqing 400016, China; 2 Department of Urology, The First Affiliated Hospital of Anhui Medical University, Hefei, China; 3 Anhui Province Key Laboratory of Genitourinary Diseases, Anhui Medical University, Hefei, China; 4 The Institute of Urology, Anhui Medical University, Hefei, China

**Keywords:** renal cell carcinoma, PKMYT1, EMT, prognosis, diagnosis

## Abstract

Clear cell renal cell carcinoma (ccRCC) is one of the most lethal urological malignancies with high tumor heterogeneity, and reliable biomarkers are still needed for its diagnosis and prognosis. WEE family kinases function as key regulators of the G2/M transition, have essential roles in maintaining cellular genomic stability and have the potential to be promising therapeutic targets in various tumors. However, the roles of WEE family kinases in ccRCC remain undetermined. In the present study, we first explored multiple public datasets and found that PKMYT1 was up-regulated in both RCC tumors and cell lines. Expression levels of PKMYT1 were highly associated with pathological stage and grade. Kaplan-Meier curves showed that high PKMYT1 expression was associated with lower overall survival and disease-free survival. Receiver operating characteristic curves revealed that the expression of PKMYT1 could better distinguish ccRCC from normal samples. Functional enrichment analysis demonstrated that cell cycle- related pathways and epithelial to mesenchymal transition (EMT) might be potential mechanisms of PKMYT1 in ccRCC tumorigenesis. Moreover, knockdown of PKMYT1 *in vitro* attenuated the proliferation, migration and invasion of RCC cell lines, promoted cell apoptosis and prevented the EMT phenotype *in vitro*. In conclusion, our study demonstrated that PKMYT1 has the potential to act as a diagnostic and prognostic biomarker for RCC patients. Targeting PKMYT1 may be considered as a new potential therapeutic method and direction in RCCs.

## INTRODUCTION

Renal cell carcinoma (RCC) is a common malignant tumor of the urinary system, it mainly originates from renal tubular epithelial cells and accounts for approximately 2% to 3% of all adult malignancies [1]. Clear cell renal cell carcinoma (ccRCC) is a common histopathologic subtype of kidney cancer, accounting for 80% to 90% of RCC [2]. Recent studies have indicated that more than 70,000 new diagnosed cases and about 15,000 deaths from kidney cancer will occur in the United States in 2020 [3]. Moreover, the incidence and mortality of ccRCC is still increasing in most countries [4]. ccRCC is the most lethal tumor, leading to the death in most RCC patients. Owing to the lack of characteristic clinical symptoms, approximately 30% of patients present with advanced disease at first visit to a doctor [5]. In addition, approximately 20–30% of patients will suffer recurrence despite successful surgical resection owing to tumor heterogeneity. Therefore, the identification of biomarkers that could facilitate patients’ exact diagnosis and distinguish patients with indolent cancer from those with aggressive cancer holds great promise.

In the cell cycle, surveillance mechanisms play vital roles in maintaining genomic integrity primarily through two DNA damage checkpoints. Most cancer types have defective G1 checkpoint mechanisms.

G1 checkpoint mechanisms exist defective to some degree in most cancer types, and that cancer cells are more sensitive to G2 checkpoint than normal cells [6]. Therefore, cell cycle G_2_ checkpoint abrogation would be a promising means to inhibit tumor proliferation [7]. WEE family kinases composed of three members (WEE1, WEE1B and PKMYT1) and can be activated as key regulators of the G2/M transition of the cell cycle, playing an important role in maintaining genome stability [7]. WEE family kinases can prevent cells from transitioning from G2 to mitosis phase in two ways [8]. One is to bind the Cdc2/cyclin B1 complex in the cytoplasm preventing entry into the nucleus [8, 9].

Alternatively, the WEE family kinases can inhibit the catalytic activity of Cdc2 by phosphorylating the Thr14/Thr15 residues [8, 10, 11]. Currently, WEE1 inhibitors have been suggested to have potential antineoplastic activity in various tumors [12–14]. Nonetheless, the mechanisms of WEE family kinases in ccRCC were not yet clear.

In the current research, we systematically investigated the functions of three members of WEE family kinases in ccRCC patients. Their expression levels, prognostic values, and relationships with clinical traits were evaluated. Moreover, we selected PKMYT1 for further investigation. The diagnostic value of PKMYT1 for tumors and its associations with clinicopathological stage were evaluated. In addition, we investigated the roles of PKMYT1 in tumorigenesis, progression and invasion and its potential functional mechanisms.

## RESULTS

### Relative expression of WEE family kinases in ccRCC

**Figure 1:**
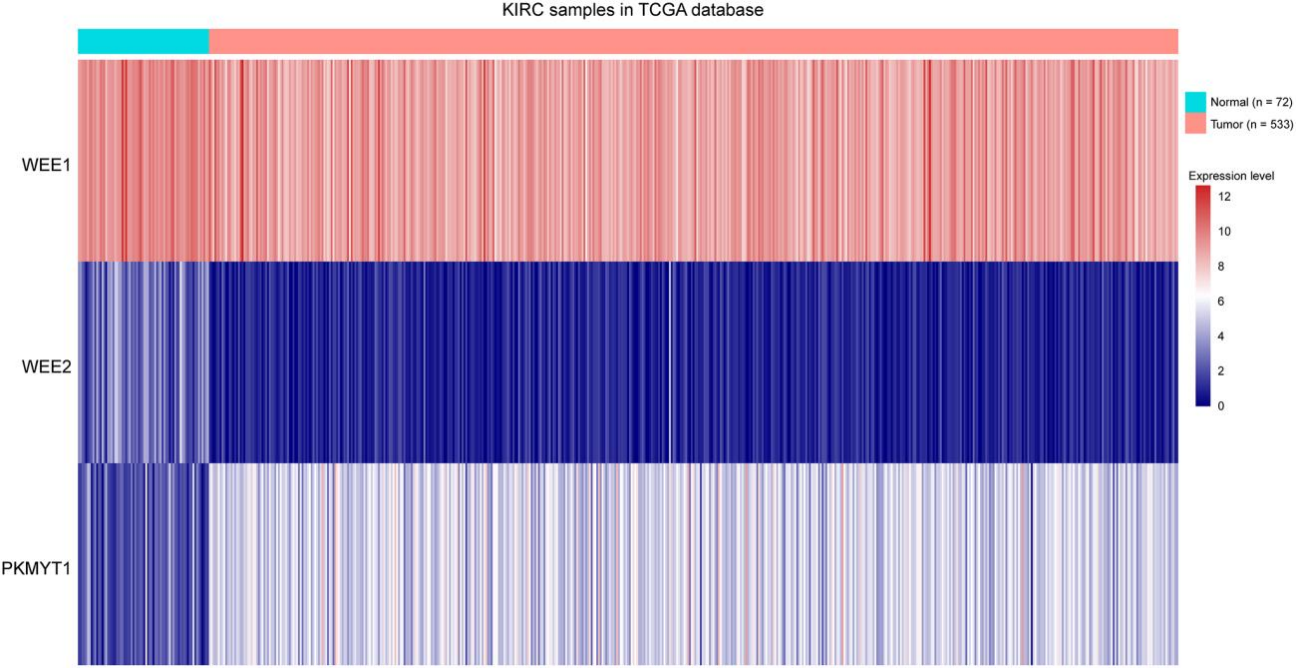
Heat map showing expression levels of three members of WEE family kinases in ccRCC tumors compared to normal tissue samples.

**Figure 2:**
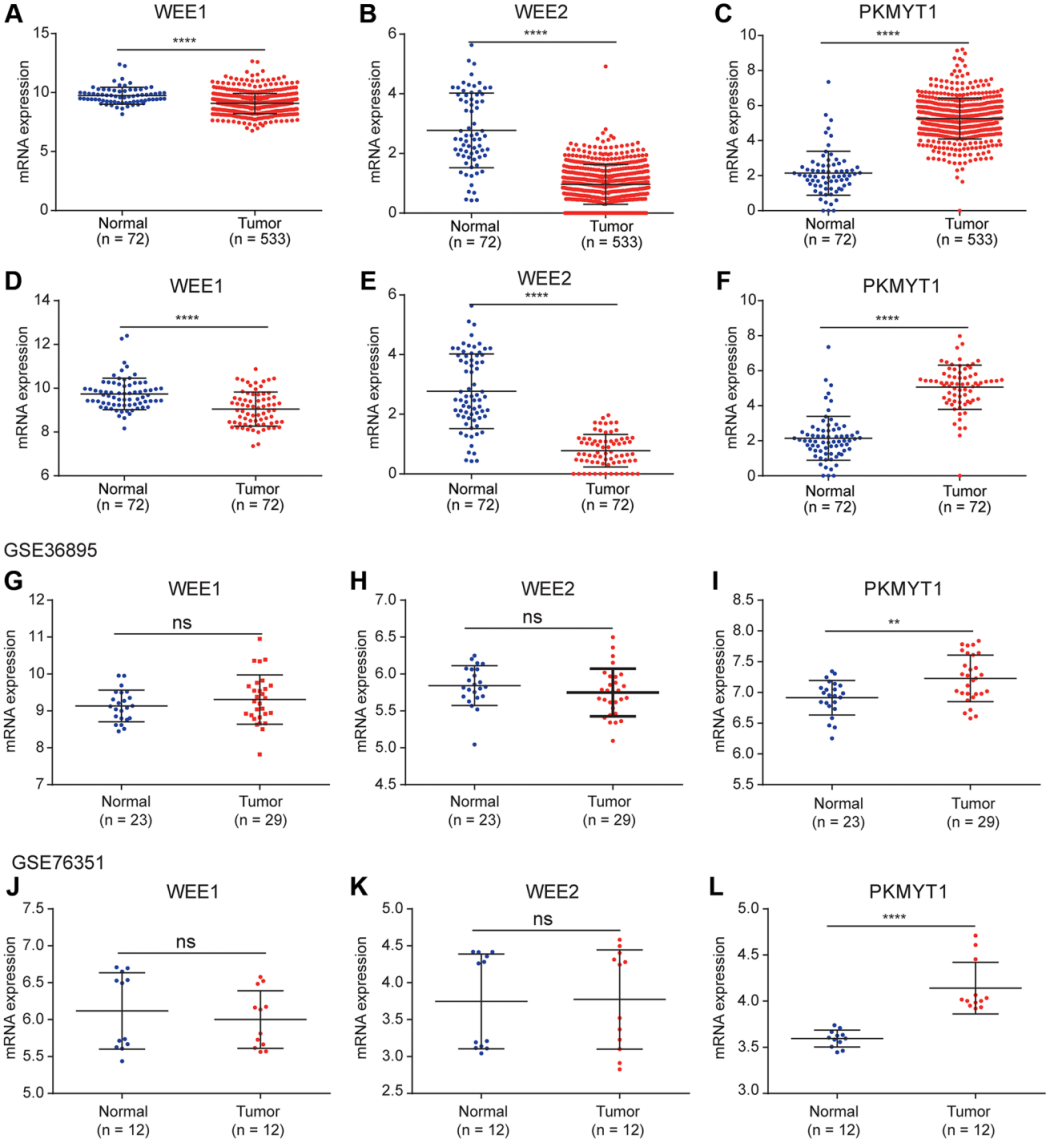
**Differential expression analysis of WEE family kinases.** Expression levels of WEE1 (**A**) and WEE2 (**B**) were down-regulated in tumors (normal (*n* = 72); tumor (*n* = 533)), while PKMYT1 (**C**) was up-regulated in tumors compared with normal tissue samples in the TCGA dataset. A consistent result for WEE1 (**D**) and WEE2 (**E**) were also obtained for paired normal and tumor samples (normal (*n* = 72); tumor (*n* = 72)), while PKMYT1 was up-regulated in tumors (**F**). Expression levels of WEE1 and WEE2 exhibited no significant difference in the GSE36895 (**G**, **H**). PKMYT1 was up-regulated in tumors in the GSE36895 (**I**). Expression levels of WEE1 and WEE2 exhibited no significant difference in the GSE76351 (**J**, **K**). PKMYT1 was up-regulated in tumors in the GSE36895 (**L**).

To explore the roles of WEE family kinases in ccRCC, we first investigated mRNA expression levels of the three members of WEE family kinases in multiple datasets. A heat map was used to illustrate the overall expression levels of the three members of WEE family kinases between normal and tumor samples in the TCGA dataset (Figure 1). Then, we compared expression levels of the three members between control and tumor samples. We observed down-regulated levels of WEE1 and WEE2 and up-regulated levels of PKMYT1 in tumors compared to normal samples for all samples in the TCGA dataset (Figure 2A–2C). We also compared expression levels of paired normal and tumor samples in the TCGA dataset, and found down-regulated levels of WEE1 and WEE2 and up-regulated levels of PKMYT1 in tumors (Figure 2D–2F). Consistently, we also compared mRNA expression levels of the three members in the GSE36895 (Figure 2G–2I) and GSE76351 datasets (Figure 2J–2L). In the validated GEO datasets, we obtained similar analytical results, in which we found up-regulated levels of PKMYT1 in tumors compared with normal samples. While, expression levels of WEE1 and WEE2 showed no significant differences between tumor and normal samples, but all results displayed overexpression of PKMYT1 in tumors.

### Prognostic values of WEE family kinases in ccRCC

**Figure 3:**
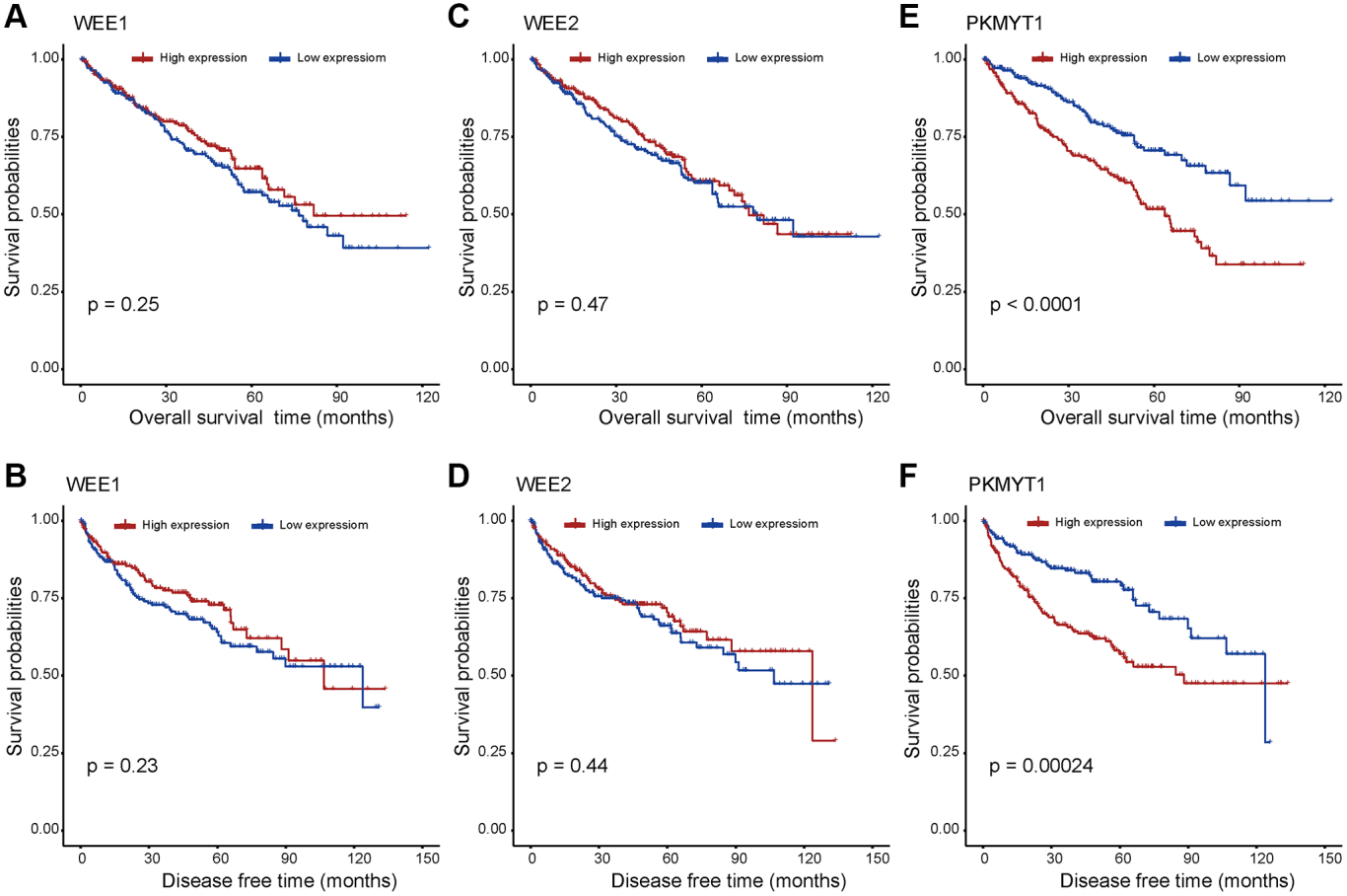
**Survival analysis for WEE family kinases.** The overall survival and disease free survival between the high and low expression groups of WEE1 (**A**, **B**) and WEE2 (**C**, **D**) displayed no significant difference. However, the high expression group of PKMYT1 tended to have lower overall survival (**E**) and disease free survival (**F**) in ccRCC.

Prognostic significance assessment of WEE family kinases in ccRCC using Kaplan–Meier curves analysis. Based on the median values of mRNA expression, we divided the patients into high and low expression groups respectively. The Kaplan–Meier curves demonstrated the overall survival of ccRCC patients, and the results reflect the poor prognosis of the PKMYT1 high expression group (Figure 3E).

However, there exhibited no significant difference for survival rates of WEE1 (Figure 3A) and WEE2 (Figure 3C) in high and low expression groups. In addition, we evaluated the disease-free survival profile of the high and low expression groups. We found that disease free survival was lower in the high PKMYT1 expression group compared to the low expression group (Figure 3F). However, the disease-free survival rate showed no significant difference for WEE1 (Figure 3B) or WEE2 (Figure 3D). Subsequently, we performed survival analysis for overall survival in different subgroups of ccRCC patients (Supplementary Figure 1). The results revealed that the high expression group presented poorer overall survival rate for the male, female, stage I + II, stage III + IV, G3+G4, higher age (>60 years) and lower age (≤60 years) subgroups. However, the survival analysis showed no significant difference in G1+G2 subgroup. Based on the above results, we hypothesized that PKMYT1 may be a prospective prognostic biomarker for ccRCC.

### PKMYT1’s association with clinical variables

**Figure 4:**
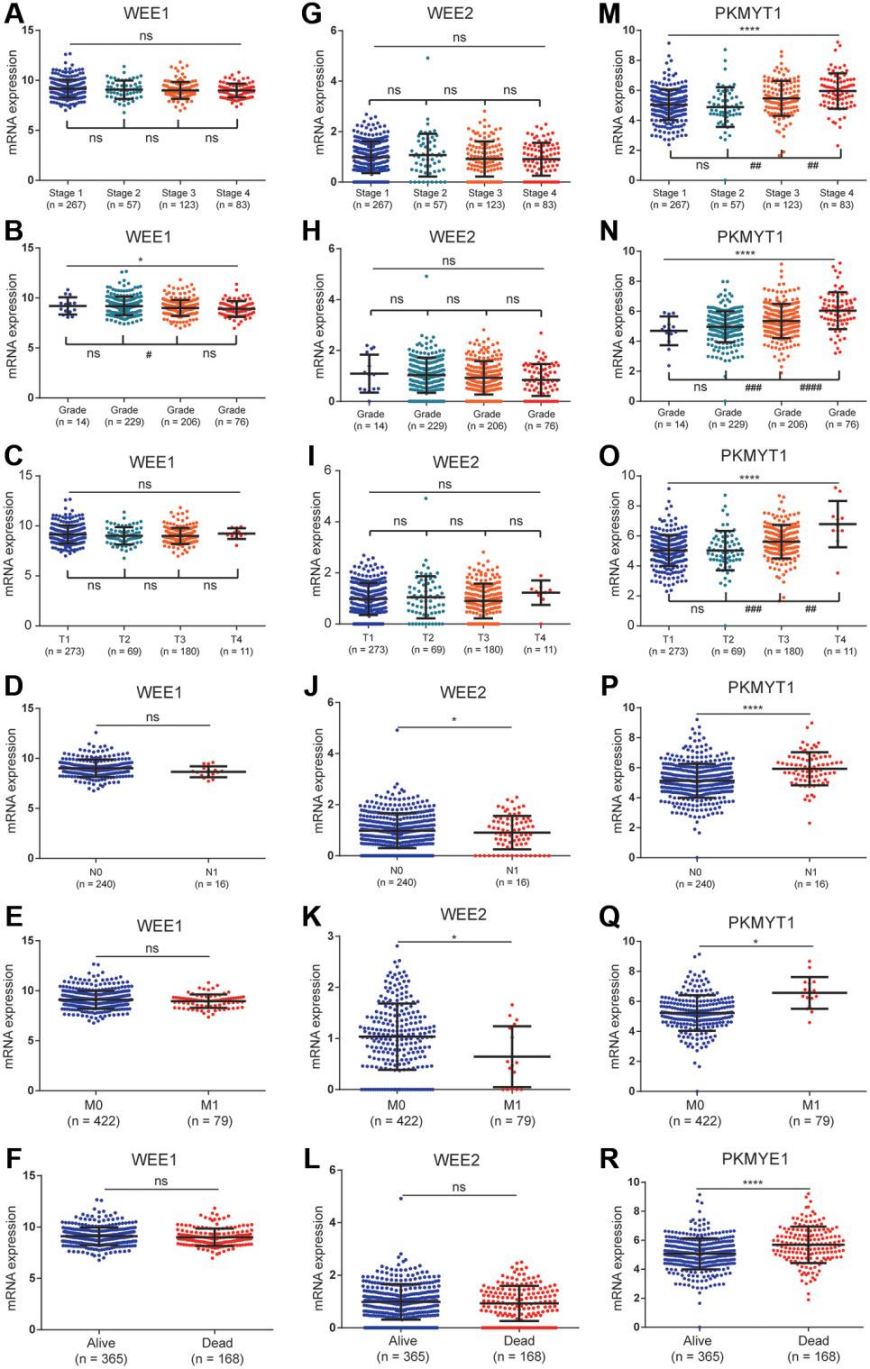
**The relationships between the expression of WEE family kinases and clinical factors.** The expression of WEE1 showed no relevance with stage (**A**), but was negatively correlated with the grade of ccRCC patients (**B**); and has no relevance to T stage (**C**), N stage (**D**), M stage (**E**), and survival status (**F**). The expression of WEE2 showed no relevance with stage (**G**), grade (**H**), T stage (**I**), but was up-regulated in the N1 (**J**) and M1 stages (**K**); and has no relevance to survival status (**L**). The expression levels of PKMYT1 were positively correlated with stage (**M**), grade (**N**), T stage (**O**), N stage (**P**), M stage (**Q**), and dead status (**R**).

The relationships between clinical variables and the expression levels among WEE family kinases were evaluated in the TCGA dataset. Expression levels of WEE1 were negatively correlated with pathological grade (Figure 4B). However, expression levels of WEE1 showed no significant differences between groups for pathological stage (Figure 4A), pathological T stage (Figure 4C), lymphatic metastasis (Figure 4D), distant metastasis (Figure 4E) or survival status (Figure 4F).

In the lymphatic metastasis (Figure 4J) and distant metastasis groups (Figure 4K), we observed a downregulation of WEE2 expression levels. However, expression levels of WEE2 showed no significant differences between groups for pathological stage (Figure 4G), pathological grade (Figure 4H) pathological T stage (Figure 4I) or survival status (Figure 4L). For PKMYT1 gene, we found significantly positive correlations between expression levels and pathological stage (Figure 4M), pathological grade (Figure 4N) and pathological T stage (Figure 4O). Additionally, we found high expression levels of PKMYT1 in lymphatic metastasis (Figure 4P), distant metastasis (Figure 4Q) and dead status (Figure 4R). These findings suggested that expression levels of PKMYT1 are highly associated with the tumor malignancy.

Taking into consideration the above results, we selected the PKMYT1 gene for further study.

**Figure 5:**
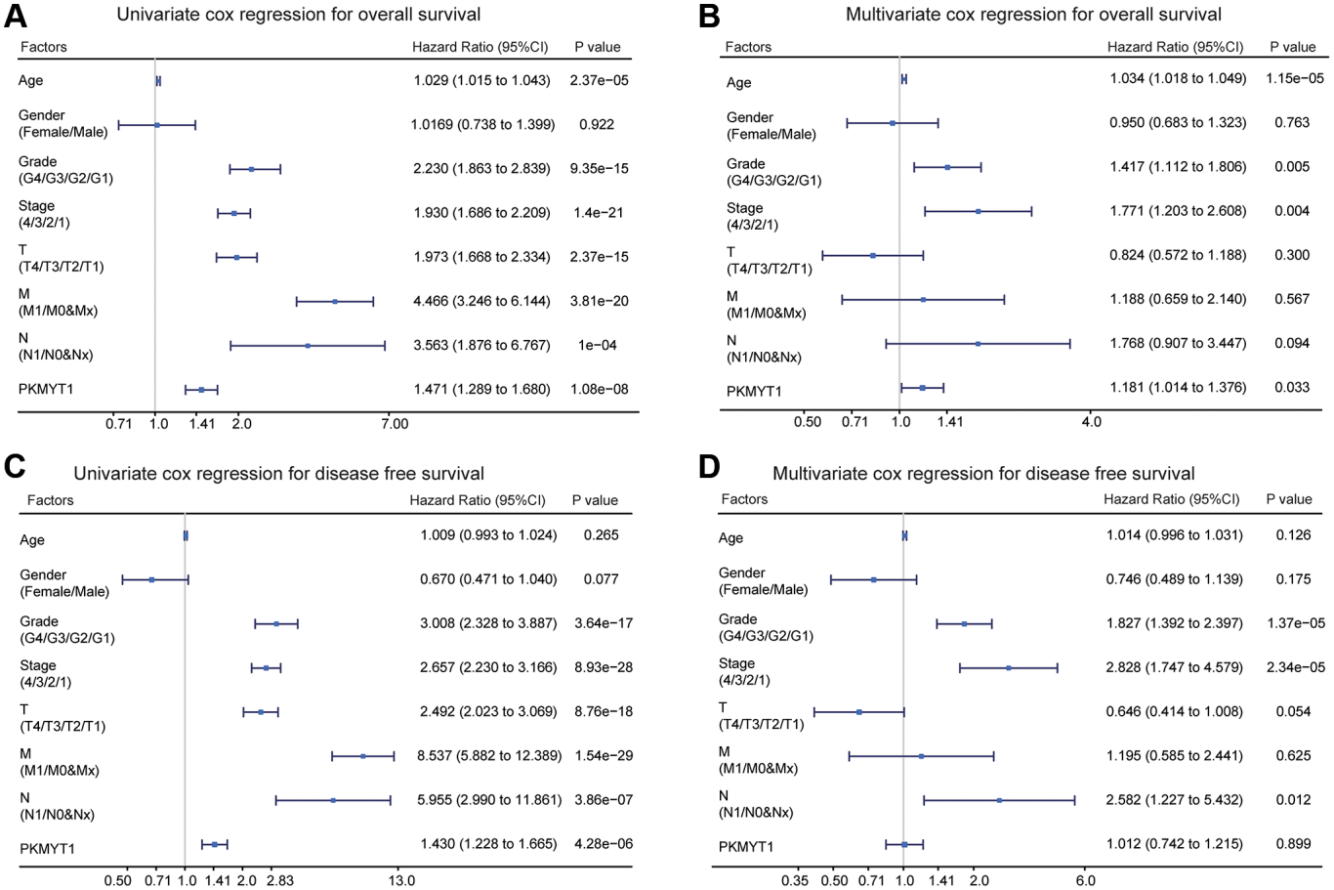
**Univariate and multivariate Cox regression analysis of overall survival and disease free survival in ccRCC patients.** (**A**) Univariate Cox regression analysis for overall survival. (**B**) Multivariate Cox regression analysis for overall survival. (**C**) Univariate Cox regression analysis of disease free survival. (**D**) Multivariate Cox regression analysis of disease free survival.

Patients were classified into high and low expression groups based on the median expression value of PKMYT1, and associations with the distributions of clinical phenotypes were investigated. The results showed that high proportions of higher T stage, distant metastasis, advanced stage, lymphatic metastasis, and higher grade in the high expression group of PKMYT1 (Table 1). However, the distributions of age and gender showed no significant difference. Furthermore, we evaluated overall survival and disease-free survival in ccRCC patients by univariate and multivariate Cox regression analysis. The results showed that PKMYT1 was a risk factor for overall survival (Figure 5A) and disease-free survival (Figure 5C) via univariate Cox regression analysis. The expression levels of PKMYT1 were a risk factor for overall survival in ccRCC patients after integration with multiple clinical factors (Figure 5B) through multivariate Cox regression analysis. However, expression levels of PKMYT1 with respect to disease-free survival showed no significant difference (Figure 5D). Based on the aforementioned findings, we confirmed that PKMYT1 is an autonomous risk factor affecting overall survival of ccRCC patients, which might provide a supplement for clinical factors.

**Table 1:** Association between PKMYT1 mRNA expression and clinical characteristics of patients with clear cell renal cell carcinoma.

**Variables**	**Number**	**PKMYT1 mRNA expression**		***P* value**
		**High (*n* = 267)**	**Low (*n* = 266)**	
Age (mean ± SD, years)	60.6 ± 12.1	60.6 ± 12.2	60.6 ± 12.1	0.908
Gender				0.079
Male	345 (64.7)	183 (53.0)	162 (47.0)	
Female	188 (35.3)	84 (44.7)	104 (55.3)	
Stage				<0.001
I	267 (50.1)	113 (42.3)	154 (57.7)	
II	57 (10.7)	20 (35.1)	37 (64.9)	
III	123 (23.0)	71 (57.7)	52 (42.3)	
IV	83 (15.6)	60 (72.3)	23 (27.7)	
Unknown	3 (0.6)	3 (100)	0 (0)	
Grade				<0.001
G1	14 (2.6)	3 (21.4)	11 (78.6)	
G2	229 (43.0)	95 (41.5)	134 (58.8)	
G3	206 (38.6)	107 (51.9)	99 (48.1)	
G4	76 (14.3)	59 (77.6)	17 (22.4)	
Unknown	8 (1.5)	3 (37.5)	5 (62.5)	
T stage				<0.001
T1	273 (51.2)	116 (42.5)	157 (57.5)	
T2	69 (12.9)	28 (40.6)	41 (59.4)	
T3	180 (33.8)	113 (62.8)	67 (37.2)	
T4	11 (2.1)	10 (90.9)	1 (9.1)	
N stage				<0.001
N0/Nx	517 (97.0)	252 (48.7)	265 (51.3)	
N1	16 (3.00)	15 (93.8)	1 (6.2)	
M stage				<0.001
M0/Mx	454 (85.2)	209 (46.0)	245 (54.0)	
M1	79 (14.8)	58 (73.4)	21 (26.6)	

### Diagnostic value of PKMYT1 mRNA expression in ccRCC

**Figure 6:**
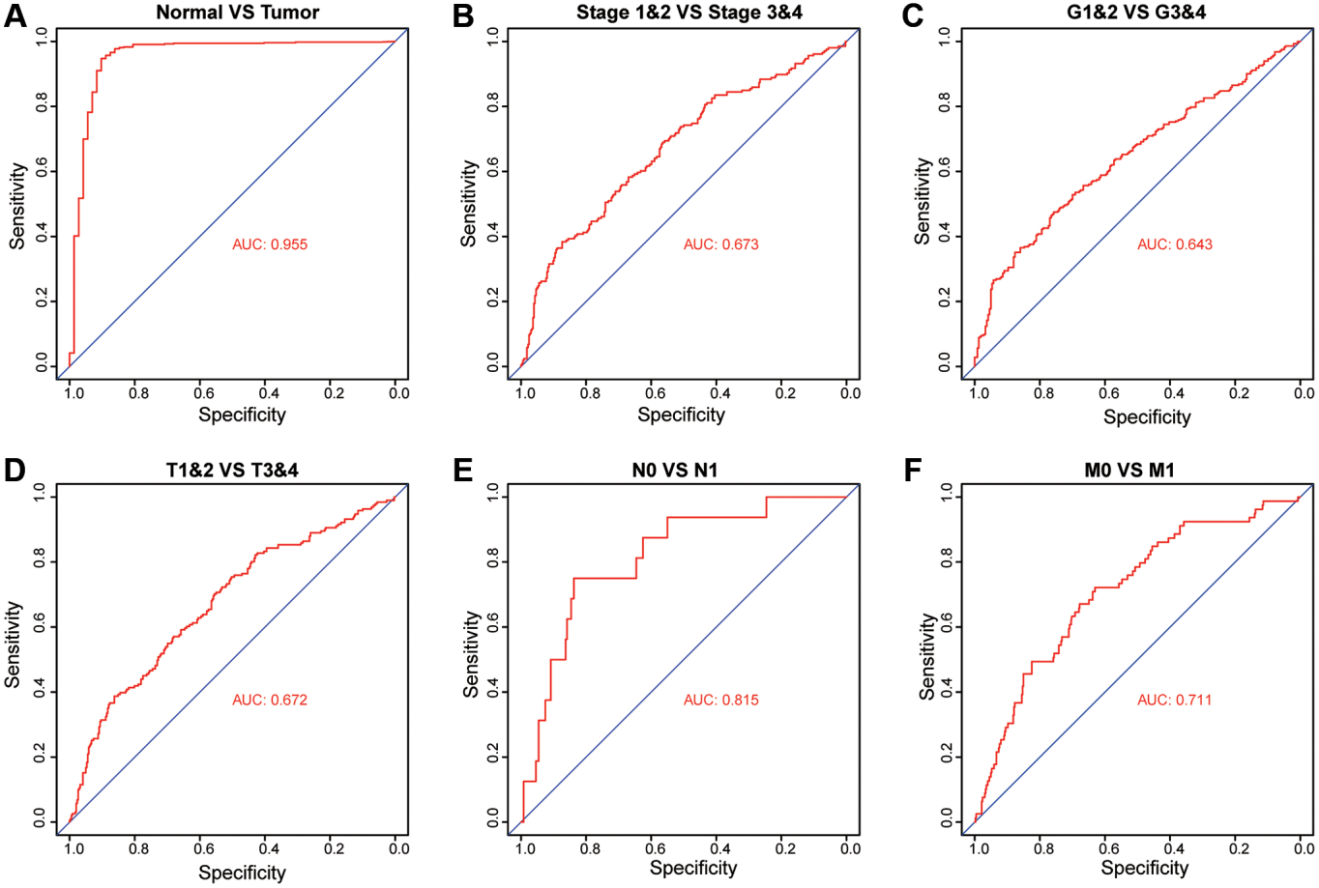
**Diagnostic value of PKMYT1 mRNA expression for ccRCC patients in the TCGA dataset.** Receiver operating characteristic curves for PKMYT1 to distinguish tumors from normal tissues (**A**), stage I + II vs. stage III + IV (**B**), G1&G2 vs. G3&G4 (**C**), T1&T2 vs. T3&T4 (**D**), N0 vs. N1 (**E**), and M0 vs. M1 (**F**).

To investigate whether the expression of PKMYT1 has diagnostic value in ccRCC patients, we performed receiver operating characteristic (ROC) investigation and calculated the area under the curve (AUC) to evaluate the diagnostic efficiency. We found that PKMYT1 sufficiently differentiated ccRCC patients from normal controls when the area under the AUC curve was 0.955 (Figure 6A). In addition, the diagnostic value of PKMYT1 expression levels were also evaluated for stage I + II vs. stage III + IV (AUC, 0.673; Figure 6B), (G1 + G2) vs. (G3 + G4) stage (AUC, 0.643; Figure 6C), (T1 + T2) vs. (T3 + T4) stage (AUC, 0.672; Figure 6D), N0 vs. N1 (AUC, 0.815; Figure 6E) and M0 vs. M1 (AUC, 0.711; Figure 6F). These findings suggest that PKMYT1 may serve as a potential diagnostic biomarker for ccRCC patients with excellent performance.

### Biological pathogenesis of PKMYT1 in ccRCC

**Figure 7:**
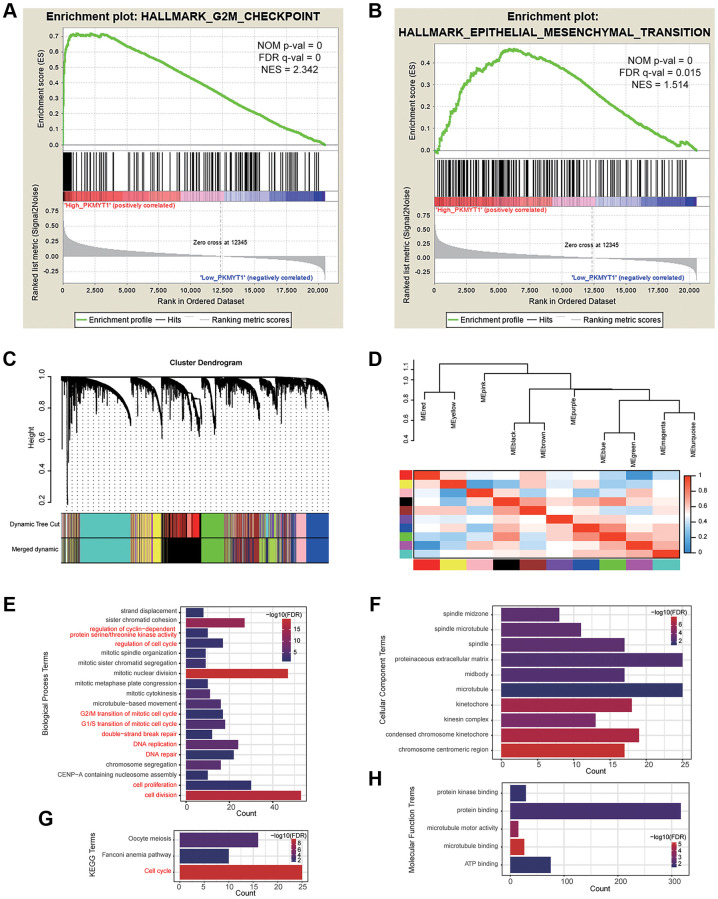
**Functional enrichment analysis.** Gene set enrichment analysis showed that high PKMYT1 expression was associated with the biological process of epithelial–mesenchymal transition (**A**) and G2M checkpoint (**B**). (**C**) The modules were identified by weighted gene co-expression network analysis. (**D**) Correlations between modules. The results of enriched terms for genes in the green module, including biological process (**E**), cellular component (**F**) molecular function (**G**) and KEGG (**H**).

To explore the functional mechanism of PKMYT1 in ccRCC, we employed GSEA analysis to uncover the statistically significant biological pathways between the high expression and low expression groups of PKMYT1 (divided by median expression level). Our findings displayed that PKMYT1expression level was connected with the biological pathways of G2/M checkpoint (Figure 7A) and EMT processes (Figure 7B). We also used the WGCNA (weighted gene co-expression network analysis) method to construct co-expression network where similarly expressed genes were merged into the same module (Figure 7C). The correlations between modules are shown in Figure 7D. We found that PKMYT1 merged into the green module, and these genes were selected for functional enrichment analysis (Supplementary Table 1). The terms, “G2/M transition of mitotic cell cycle”, “regulation of cell cycle”, “G1/S transition of mitotic cell cycle”, “regulation of cyclin-dependent protein serine/threonine kinase activity” and “cell proliferation” were enriched for biological process (Figure 7E). The terms “chromosome, centromere region” and “condensed chromosome kinetochore” were enriched for cellular component (Figure 7F). The terms, including “protein binding”, “protein kinase binding” and “ATP binding” were enriched for molecular function (Figure 7H). The cell cycle term was enriched according to KEGG analysis (Figure 7G). Based on these results, we deduced that the cell cycle related pathway and EMT might be the potential mechanisms of PKMYT1 in ccRCC tumorigenesis.

### High expression of PKMYT1 in ccRCC cells and tumor tissues

**Figure 8:**
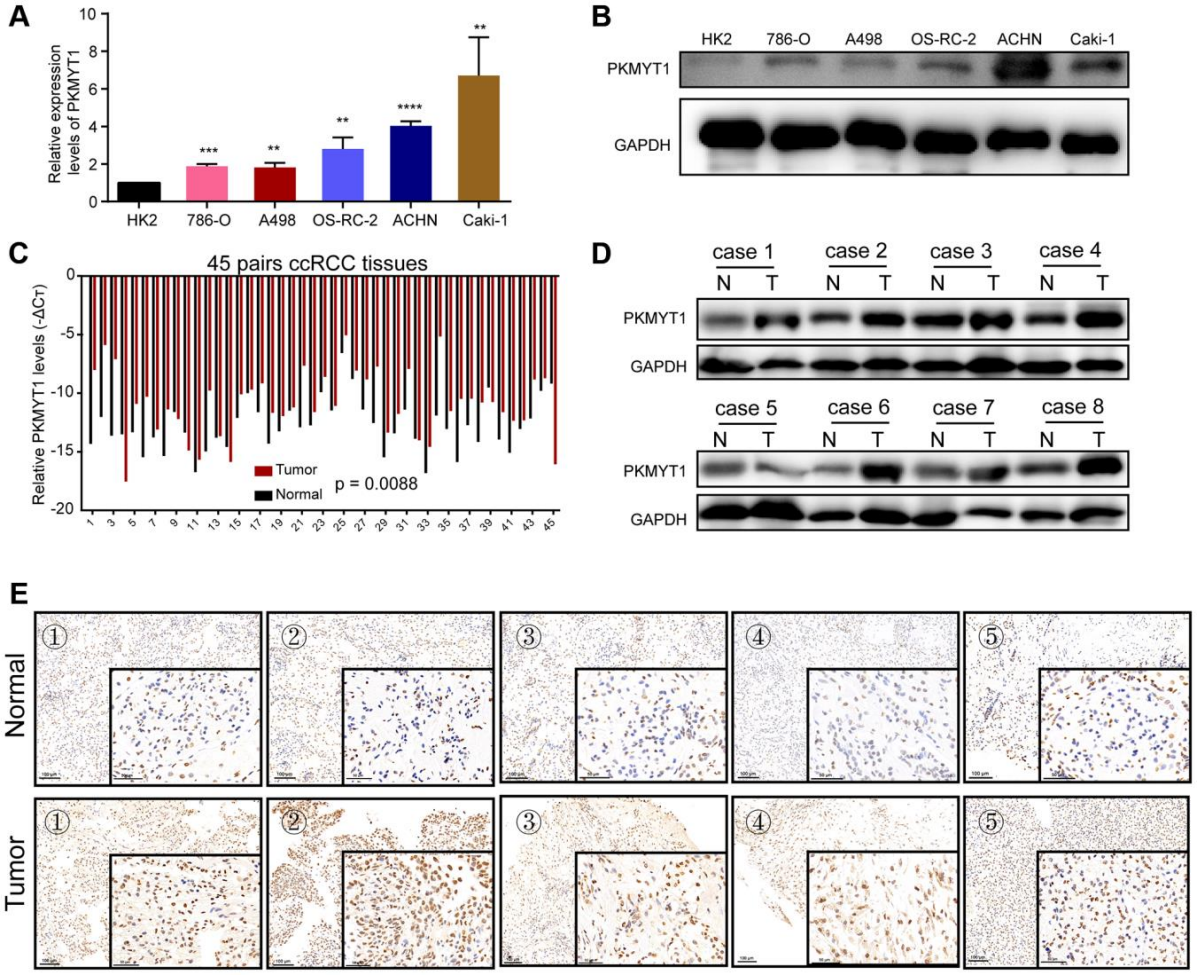
**Overexpression of PKMYT1 in renal cancer cells and tissues.** The expression of PKMYT1 in renal cancer cells was measured using real-time quantitative reverse transcription-PCR (**A**) and western blotting assays (**B**). The expression of PKMYT1 in renal cancer tissues and paired normal tissues was measured using real-time quantitative reverse transcription-PCR (**C**), western blotting assays (**D**) and immunohistochemistry (**E**). Data are presented as the mean ± standard deviation of three independent experiments, and were compared to HK-2 cells. ^**^P < 0.01, ^***^P < 0.001; ^****^P < 0.0001.

To detect PKMYT1 expression levels in ccRCC cells and tumor tissues, qRT–PCR and western blotting assays were executed. Compared to HK-2, mRNA expression levels (Figure 8A) and protein levels (Figure 8B) of PKMYT1 were upregulated in ccRCC cells, including ACHN, Caki-1, A498, OSRC-2, and 786-O cell lines. The expression of PKMYT1 in paired tissues of ccRCC patients was also evaluated. The results showed that higher PKMYT1 expression in comparison with the paired normal neighboring tissues (Figure 8C). In contrast, our western blotting assay (Figure 8D) and immunohistochemical analysis (Figure 8E) both confirmed the increased protein expression level of PKMYT1 in ccRCC tissues.

### Knockdown of PKMYT1 significantly inhibits the cell migration, proliferation, and invasion of ccRCC

**Figure 9:**
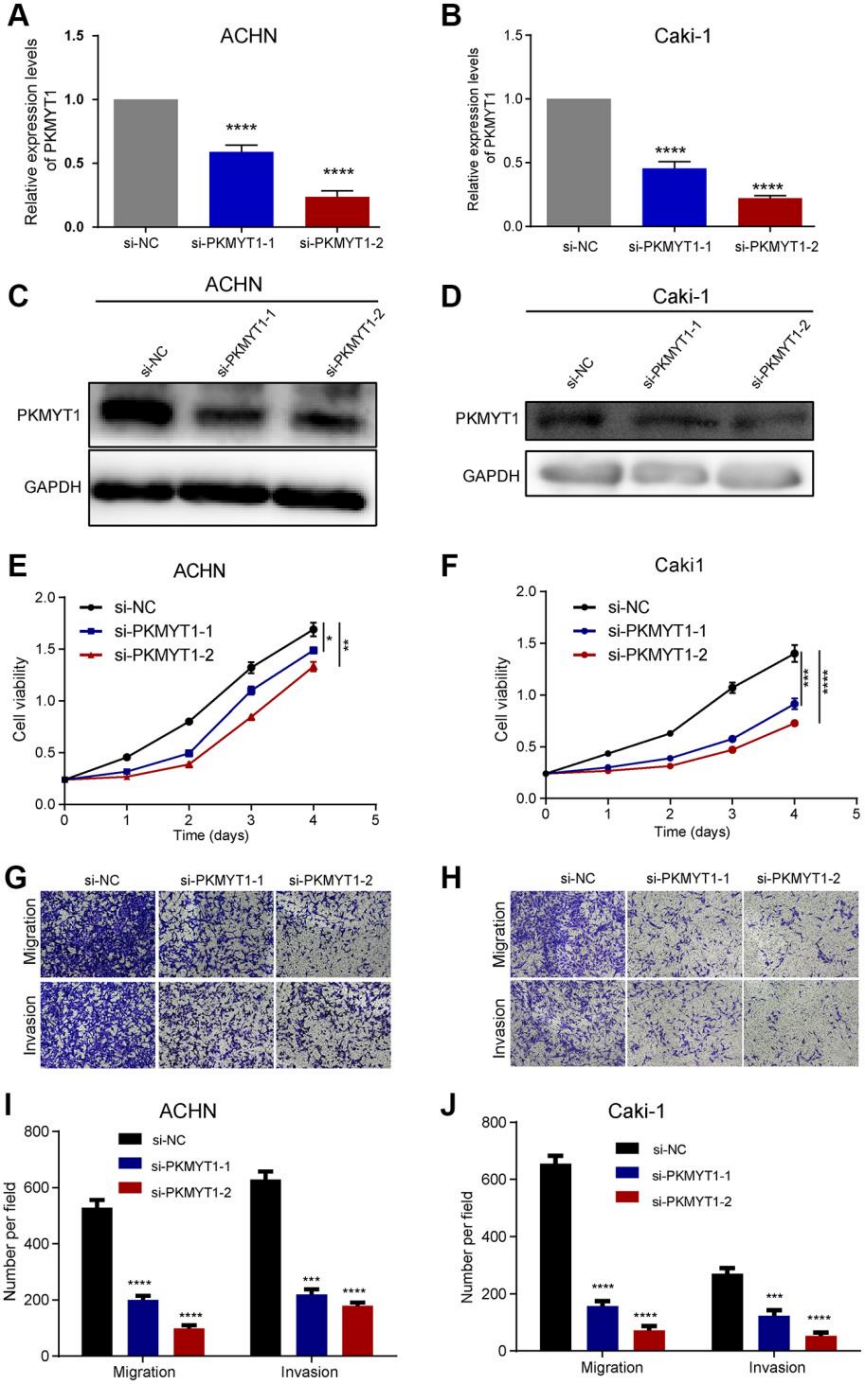
**PKMYT1 promotes the proliferation, migration, and invasion of renal cancer cells *in vitro*.** The mRNA expression of PKMYT1 in ACHN (**A**) and Caki-1 cells (**B**) was measured using real-time quantitative reverse transcription-PCR. The protein expression of PKMYT1 in ACHN (**C**) and Caki-1 cells (**D**) by western blotting assays. Cell Counting Kit-8 assays were used to detect the effect of PKMYT1 knockdown on cell proliferation in ACHN (**E**) and Caki-1 (**F**) cells. Representative images of migration and invasion assays for ACHN (**G**) and Caki-1 (**H**) cells. Transwell migration and invasion assays indicated that the migration and invasion abilities of PKMYT1 were weakened in siRNA groups for ACHN (**I**) and Caki-1 (**J**) cells. Data are shown as the mean ± standard deviation from three independent experiments, and were compared to the respective si-NC group. ^****^P < 0.0001; ^***^P < 0.001; ^**^P < 0.01 and ^*^P < 0.05.

To examine the effect of PKMYT1 in the biological medicine of ccRCC, we transfected si-PKMYT1 into RCC cell lines (ACHN and Caki-1) by down-regulated its expression levels. The down-regulated mRNA levels of PKMYT1 were confirmed using qRT-PCR for ACHN cells (Figure 9A) and Caki-1 cells (Figure 9B) compared to the corresponding negative control. Down-regulated protein levels of PKMYT1 were confirmed using western blotting assays for ACHN cells (Figure 9C) and Caki-1 cells (Figure 9D) compared to the corresponding negative control. Subsequently, whether PKMYT1 gene has an effect on cell migration, proliferation and invasion, a series of assays were performed.

The results of cell proliferation analysis manifested that knockdown of PKMYT1 uncommonly inhibited cell proliferation ability of cells in both ACHN cell lines (Figure 9E) and Caki-1 cell lines (Figure 9F) compared to the negative control. Next, we wanted to know whether knocking down PKMYT1 has any effect on the invasion and migration ability of ccRCC cells? Therefore, we used Transwell’s experiment to further evaluate. Through cell migration and invasion assays, we found that knockdown of PKMYT1 gene expression significantly debilitated the migration and invasion ability of ACHN cells (Figure 9G, 9I) and Caki-1 cells (Figure 9H, 9J). Overall, the multifaceted experiments demonstrated that PKMYT1 contributes to the migration, proliferation and invasion of ccRCC cells.

**Figure 10:**
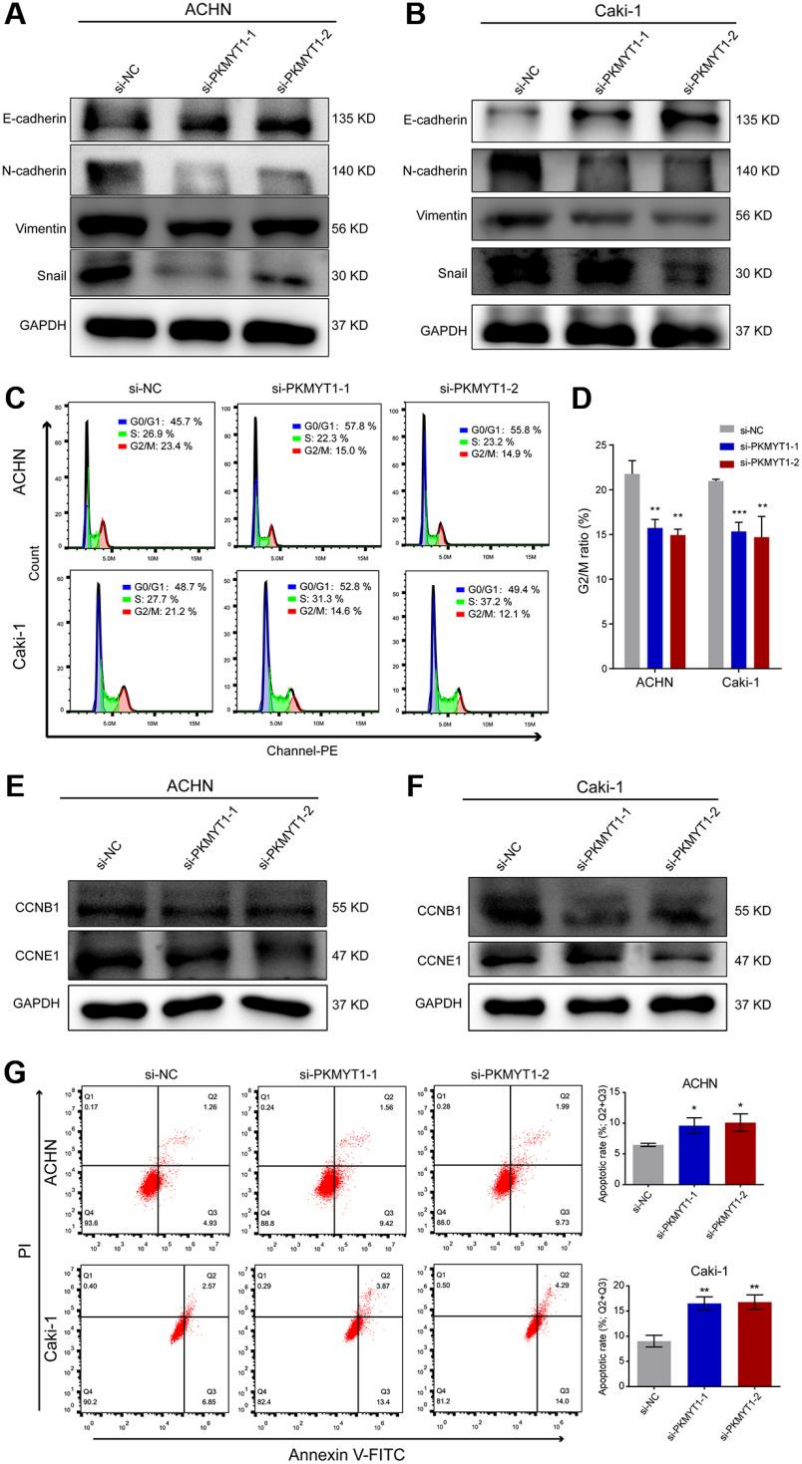
**Downregulation of PKMYT1 inhibits epithelial-mesenchymal transition (EMT), promotes apoptosis, and decreases the G2/M ratio in the cell cycle.** (**A**–**B**) Western blot analysis showing changes in EMT-related biomarkers in the ACHN and Caki-1 cell lines. Expression of E-cadherin was increased in the siRNA groups, while expression of N-cadherin, Vimentin, and Snail was decreased. (**C**) Representative images of flow cytometry results for the cell cycle in ACHN and Caki-1 cells. (**D**) Results showing reduction of G2/M ratio in cell cycle of ACHN and Caki-1 cells transfected with PKMYT1 siRNA versus a negative-control siRNA. (**E**–**F**) Western blot analysis of the protein expression levels of CCNB1 and CCND1 in ACHN and Caki-1 cell lines transfected with a negative-control siRNA or PKMYT1 siRNA. (**G**) Flow cytometry showing that inhibiting the expression of PKMYT1 contributed to a high apoptosis rate in ACHN and Caki-1 cells. ^*^P < 0.05; ^**^P < 0.01; ^***^P < 0.001.

To further shed light on the molecular mechanism how PKMYT1 affects the metastatic capability of ccRCC cells, we assessed the EMT phenotype, which plays crucial roles in tumor progression and is intently related to cell invasion and migration. Therefore, we examined the expression of some important protein markers during EMT, such as Vimentin, N-cadherin, E-cadherin and Snail by western blot assays. As shown in Figure 10A, knockdown of the PKMYT1 reduced the expression of mesenchymal-associated protein markers (N-cadherin, Vimentin and Snail) and conversely upregulated the protein levels of epithelial markers (E-cadherin) in ccRCC cells. We carried out the same experiments in the Caki-1 cell line following PKMYT1 knockdown and obtained similar experimental findings (Figure 10B). These results all indicated that PKMYT1 accelerates ccRCC cells migration and invasion through EMT.

### Implication of PKMYT1 knockdown on the cell apoptosis and cell cycle

Based on the functional enrichment analysis, we speculated that the cell cycle-related pathway might underlie the potential functional mechanism of PKMYT1. Therefore, we designed two different siRNAs and subsequently transfected into ccRCC cells to knock down the expression of PKMYT1 gene. Based on flow cytometry experiments to detect the cell cycle, our results exhibited that the proportion of cells in G2/M phase was reduced in ACHN and Caki-1 cell lines when transfected with PKMYT1 siRNA (Figure 10C, 10D). The previous GSEA analysis revealed that PKMYT1-interplaying proteins are closely associated with cell cycle progression. Then we explored the protein expression of indispensable cyclin genes such as CCNB1 and CCNE1 in ccRCC cells. The results showed that all of these cell cycle-associated proteins were reduced in the PKMYT1 knockdown groups (Figure 10E, 10F). Taken together, these findings indicated that PKMYT1 promotes cell proliferation by affecting the cell cycle. Additionally, to investigate the influence of PKMYT1 on ccRCC cell apoptosis, flow cytometry analysis was performed. We observed that the apoptosis rate of the siRNA groups in ACHN and Caki-1 cell lines was higher than negative control group (Figure 10G).

## DISCUSSION

As one of the most lethal urological malignancies, ccRCC has a high degree of tumor heterogeneity [15] and the recurrence rate of the patient is still up to 40% after radical nephrectomy [16]. Therefore, identification of potential biomarkers with diagnostic and prognostic value is essential, as they are expected to be therapeutic targets for ccRCC and provide an alternative perspective for understanding the underlying mechanisms of ccRCC development. In the current research, we first used bioinformatics methods to systematically explore the functions of WEE family kinases in ccRCC through multiple public databases. PKMYT1 as a member of the WEE family, which may have important roles in ccRCC patients. At present, several studies have reported some degree of variation about the PKMYT1 gene in the development and progression of multiple tumor, including esophageal squamous cell carcinoma [17], prostate cancer [18], breast cancer [19], liver [20] and colorectal carcinomas [21]. Then, the diagnostic and prognostic values, roles in tumorigenesis, progression and invasion, and potential functional mechanisms of PKMYT1 were systematically investigated.

During double-stranded DNA breaks, most cancer cells are highly dependent on the G2 checkpoint of the cell cycle to detect, owing to inherent defects in the of G1 checkpoint in cancers [6, 7, 22]. WEE family kinases function as key regulators of the G2/M transition, which have essential roles in maintaining cellular genomic stability and have the potential to be promising therapeutic targets in various tumors [7]. However, their performances in the ccRCC have not yet been determined. In the present research, the mRNA expression levels of three members of the WEE family kinases in ccRCC were investigated for the first time. We found that in the WEE family, only the PKMYT1 gene was up-regulated and over-expressed in ccRCC. While, the expression of WEE1 and WEE2 tended to be downregulated. In addition, we also discovered a positive correlation between expression levels of PKMYT1 and the malignancy of ccRCC patients. Survival analysis showed that the PKMYT1 high expression subgroup had a lower survival rate. Multivariate Cox regression analysis suggested that PKMYT1 could function as a self-reliant risk factor for the survival of ccRCC patients. All these results all support the notion that PKMYT1 may serve as a promising prognostic biomarker for ccRCC patients.

PKMYT1, a cell cycle-regulated kinase, has been identified as an oncogene driving the development of various tumors. Liu et al. showed that PKMYT1 promotes growth, migration and metastasis by triggering the β-catenin-mediated TCF signaling in hepatocellular carcinoma [20]. Wang et al. found that PKMYT1 promotes the proliferation of prostate cancer by anchoring the expression of CCNB1 and CCNE1, and fostamatinib as an antagonist of PKMYT1 could energetically inhibited proliferation, suggesting that PKMYT1 may act as a potential curative target in prostate cancer [18]. Jeong et al. showed that PKMYT1 is associated with the development of colorectal cancer [21]. Long et al. exhibited that knockdown of PKMYT1 improved the response to radiation therapy for lung adenocarcinoma by eliminating the G2/M arrest [23]. Sun et al. found that in non-small cell lung cancer, PKMYT1 plays an important role in tumorigenesis and proliferation through the Notch signaling pathway [24]. Nevertheless, the functions of PKMYT1 in ccRCC remain ambiguous. Taking into account the overexpression of PKMYT1 in tumors and its prognostic significance, we selected PKMYT1 for further study to elucidate the underlying molecular mechanism.

The relationships between the expression of PKMYT1 and clinicopathological factors were investigated in the current investigation. The research revealed that high PKMYT1 expression correlated significantly with higher stage, grade and the stage of tumor, regional lymph nodes, and distant metastasis. ROC curves exhibited good performance of diagnostic efficiency, indicating that PKMYT1 is promising as a diagnostic biomarker in ccRCC patients. In addition, we further demonstrated overexpression of PKMYT1 in both RCC cell lines and tumor samples. To explore the possible mechanism effect of PKMYT1 in ccRCC, we used siRNA to downregulate PKMYT1 expression *in vitro* and found that downregulation of PKMYT1 could weaken cell migration, invasion, and proliferation. A wealth of studies have confirmed that aberrant apoptosis is correlated with the initiation and progression of multiple types of cancers [25]. Our findings demonstrated that PKMYT1 silencing significantly enhanced apoptosis and inhibited ccRCC cell proliferation, consistent with previous studies [17, 26]. Therefore, we speculate that PKMYT1 might act as an oncogene in ccRC and its inhibition may play an antitumor role by inducing apoptosis, suggesting potential therapeutic avenues for the treatment of ccRCC for future study.

Re-activation of the epithelia mesenchymal transition in cancer cells acquires some phenotype attributes, for instance resistance to anoikis, migration, chemical resistance, invasion and tumor- triggering potential [27, 28]. Previous studies have shown that PKMYT1 positively regulates EMT in hepatocellular carcinoma cells [20]. In the present research, GSEA analysis demonstrated that high PKMYT1 mRNA level was associated with the biological process of EMT and the G2/M checkpoint. Therefore, we hypothesized that PKMYT1 promotes tumor progression by regulating EMT, and cell cycle progression in ccRCC. EMT is recognized as the most significant incidents in tumor metastasis -related processes. We found that relevant biomarkers of EMT have changed. In our assays, knockdown of the PKMYT1 gene resulted in aberrant variation of several biomarkers during EMT, for instance, upregulation of E-cadherin and downregulation of mesenchymal markers (Vimentin, N-cadherin and Snail), which was also in line with previous studies [17, 21, 29]. These findings enhance our understanding that PKMYT1 may play an indispensable role in EMT of ccRCC cells according to these associated molecular networks. In addition, genes with similar expression patterns may have the same functions; thus, we used WGCNA methods to identify genes highly correlated with the expression of PKMYT1. Through functional enrichment analysis, our results indicated that these genes are enriched in cell cycle related pathways. The cell cycle consisted of a sets of systematic processes of cell division and proliferation, including cyclins and cyclin-dependent kinase (CDK) complexes [30]. Early studies indicated that PKMYT1 inhibits cell cycle routines via suppressing the phosphorylation activity of cyclin-dependent kinase. We experimentally demonstrated that PKMYT1 influences the cell cycle progression of ccRCC by interacting with CCNB1 and CCNE1.

Reviewing all our analytical studies, we mainly conduct an investigation into the roles of PKMYT1 in ccRCC progression. We found PKMYT1 upregulation both in cell lines and tissue samples of ccRCC. More importantly, we revealed good diagnostic and prognostic values of PKMYT1 for ccRCC patients by combining multiple bioinformatics methods such as differential gene expression analysis correlated with clinicopathological features, weighted correlation-network and univariate and multivariate analyses. Therefore, PKMYT1 has the potential to be a diagnostic and prognostic biomarker in ccRCC patients. The expressions of PKMYT1 were highly correlated with the clinicopathologic features of ccRCC patients. Downregulation of PKMYT1 significantly inhibit cell migration, proliferation and invasion of ccRCC, promoted cell apoptosis, and restricted the EMT phenotype *in vitro*. All of these discoveries provide fresh perception for understanding the potential role of PKMYT1 in ccRCC, and indicated a new therapeutic strategy for ccRCC patients.

## MATERIALS AND METHODS

### ccRCC patient tissue samples

45 pairs surgical specimens of ccRCC and adjacent normal renal tissues were collected from the Department of Urology, The First Affiliated Hospital of Anhui Medical University (Hefei, China) between May 2019 and March 2021. Adjacent normal control samples were acquired ≥2 cm away from the tumor site. Samples were freshly frozen in liquid nitrogen and then placed in ultra-low temperature refrigerator (−80°C). Two pathologists uniformly confirmed the histological diagnosis type of ccRCC in these surgical specimens. Informed consent forms were signed by all patients. The present study was approved by the Ethics Committee of Human Research of The First Affiliated Hospital of Anhui Medical University (No. PJ2019-14-22).

### RNA extraction and qRT-PCR

Tissue total RNA was obtained with the TRIzol Reagent BD (Invitrogen, Carlsbad, CA). The concentration as well as purity of the RNA solution were measured using a NanoDrop 2000 spectrophotometer (NanoDrop Technologies, Wilmington, DE, USA). Based on the manufacturer’s instructions, 2 μg RNA was utilized for reverse transcription by applying a PrimeScriptTM RT reagent kit (Takara, Kusatsu, Japan). Then, the cDNA was subjected to qPCR with the SYBR Green Mix (Takara, Kusatsu, Japan). We performed the amplification reaction on an ABI7500 platform (Thermo, Massachusetts, USA). GAPDH as an internal reference gene to normalize the relative expression of PKMYT1using the 2^−ΔΔCT^ formula. The primers were chemically synthesized by Sangon Biotech (Sangon, Shanghai, China). The gene primers were presented as follows: PKMYT1, (forward) 5′-CATGGCTCCTACGGAGAGGT-3′ and (reverse) 5′-ACATGGAACGCTTTACCGCAT-3′; GAPDH, (forward) 5′-GGGAGCCAAAAGGGTCAT-3′ and (reverse) 5′-GAGTCCTTCCACGATACCAA-3′.

### Western blotting assay

For western blot analysis, cells were harvested by using radio-immunoprecipitation assay (RIPA) buffer (Beyotime Biotech, Jiangsu, China) with 1 mM inhibitor cocktail (eg: protease and phosphatase) and 1 mM PMSF. PVDF membrane (Millipore) and SDS-PAGE gel were prepared in advance for subsequent experiments. The antibody reagents used in this study were as follows: anti-E-cadherin (Santa Cruz), anti-N-cadherin (Santa Cruz), Snail (Proteintech), Vimentin (Proteintech), PKMYT1 (CST), CCNB1 (Boster), CCNE1 (Proteintech) and GAPDH (CST). GAPDH was detected as a loading control. A Fujifilm LAS4000 mini luminescent image analyzer was performed to photograph the blots.

### Immunohistochemistry

The clinically collected renal clear cell carcinoma tissues were embedded in paraffin and made into tissue sections. Subsequently, the tissue sections were dewaxed, antigen repaired, blocked, incubated with primary antibodies, incubated with secondary antibodies, DAB color development, and blocked.

Briefly, 4 μm thickness paraffin-embedded tissue sections were subjected to high-temperature antigen retrieval for 10 minutes in 0.01M citric acid buffer (pH 6.0) and then naturally cooled to room temperature. Endogenous peroxidase was removed with 3% H2O2, 0.3% Triton permeabilization, followed by blocking with 10% bovine serum albumin for 30 min. Then slides were incubated overnight at 4°C with primary antibodies targeting PKMYT1 at a 1:50 dilution. The primary antibody was then washed off with PBS, following the secondary antibody was added dropwise and incubated for 1 h at room temperature. Finally, DAB (daiminobenezidine) (ZSBio) was used for sections visualization and nuclei were re-stained with Mayer-modified hematoxylin.

### Cell culture and transfection

The 786-O, A498, OSRC-2, ACHN, Caki-1, and HK-2 cells were acquired from the cell culture center of the Chinese Academy of Medical Sciences (Shanghai, China). Except for the ACHN cell lines were cultured in MEM medium (Gibco), the remaining cell lines were maintained in DMEM/high glucose medium (Gibco) supplemented with 10% fetal bovine serum and 1% penicillin-streptomycin solution. All cell lines were cultured in a cell culture incubator at 37°C with 5% CO_2_. The medium was changed daily with fresh medium and cell passages were performed when cells reached 70% growth. Two interference sequences that specifically target the PKMYT1 gene (siRNA#1: 5′-CTGGAAGTGGCATGCAACA-3′ and siRNA#2: 5′-GCGGTAAAGCGTTCCATGT-3′) and a negative control (si-NC) siRNA (Contract number: P202101170036) were synthesized by RiboBio Co., Ltd (Guangzhou, China). According to the manufacturer’s protocol, renal cancer cell lines were transfected with si-PKMYT1 (60 nM) or si-NC (60 nM) via Lipofectamine 2000 (Invitrogen), and then added to the culture medium at a final concentration of 100 nM.

### Cell proliferation assay

For the cell proliferation assay, the 96-well plates were incubated at 37°C with 1000 cells per well, and five replicate wells and blank control wells (200 μl of FBS-free medium only) were set up for each group. Take out the 96-well plate every day, discard the old medium and add fresh medium to each well, and then add 10 μl of CCK8 (Dojindo Molecular Technology) reagent to each well in the dark.

The OD450 values of each well were measured every 24 hours for a total of 4 days using an enzyme-linked immunosorbent assay (ELISA). We finally displayed the number of cells per day in the form of a line graph to reflect the rate of cell proliferation.

### Cell cycle analysis

After cell transfection, cells were washed with PBS (centrifuged at 2000 rpm, 5 min) to collect and adjust the cell concentration to 1 × 10^6^/ml, and 1 ml of single cell suspension was taken for subsequent analysis. According to the manual instructions of the cell cycle detection kit (KeyGEN BioTECH, KGA512), we detected the cell cycle by flow cytometry. All data were analyzed by FlowJo software, V7.6.

### Apoptosis assay

Cells grown in the logarithmic phase were seeded in 24-well plates with 100 × 103 cells per well, and the cells are collected after 24 hours of cultivation until the cell concentration was about 80%–90%. Add 5 μL Annexin V-FITC (DOJINDO), gently pipette evenly and incubate at room temperature for 10 min; then add 10 μL PI dye solution (20 μg/ml) with incubating at room temperature for 5 min in the dark. Finally, flow cytometer was used to detect the cells and Flowjo software (Tree Star Software, San Carlos) was employed to visualize the results.

### Transwell cell migration and invasion assays

Cell migration and invasion assays were executed in a 24-well Transwell chamber with 8 μm pores (Costar, Bodenheim, Germany). PKMYT1 siRNAs were transfected into ACHN and Caki-1 cells before subsequent experimentation. ACHN and Caki-1 cells were incubated in serum free medium for 8 h. For migration assays, lower chamber (i.e. bottom of 24-well plate) were added 600 μ1 medium containing 10% FBS and 200 μ1 cell suspension to the upper chamber and continue incubation in the incubator for 24 h. For invasion assays, the upper wells were coated in Matrigel (diluted in serum-free medium 1:8; BD Biosciences), and incubated at 37°C for 8 h. Subsequently, add 600 μ1 of medium containing 10% FBS to the lower chamber of the 24-well plate, then place the Transwell chamber in the 24-well plate with tweezers, add 200 μ1 of the cell suspension to the upper chamber, and finally place it in the incubator for 24 h. After 24 hours of incubation, take out the cell, aspirate the medium, and gently wipe the cells in Matrigel and the upper chamber with a cotton swab. Then fixed with 4% paraformaldehyde for 15 minutes, and stained with 0.05% crystal violet for 5–10 minutes.

### Bioinformatics analysis

The mRNA expression data and clinical information (stage, grade, overall survival, status and T, N, M categories) of three members of WEE family kinases in the TCGA dataset were obtained from UCSC Xena (https://xenabrowser.net/). All expression data uses RSEM algorithm to normalize the expression of different genes. Information on the disease-free survival of ccRCC was acquired from the cBio Cancer Genomics Portal (http://cbioportal.org). The normalized independent microarray datasets, namely, GSE36895 (normal = 23; tumor = 29) and GSE76351 (normal = 12; tumor = 12), were obtained from the Gene Expression Omnibus (GEO; https://www.ncbi.nlm.nih.gov/geo/) database. GEO dataset (GSE36895) was sequenced applying the Affymetrix Human Genome U133 Plus 2.0 Array platform (GPL570), and the probes were annotated employing the corresponding “hgu133plus2.db” R package. The GSE76351 dataset was sequenced by the Affymetrix Human Gene 1.1 ST Array platform (GPL11532). For the annotation of the probes, we use the corresponding "hugene11sttranscriptcluster.db" R package to carry out. Normalization of expression data in the GSE36895 and GSE76351 datasets according to robust multi-array averaging (RMA) algorithm. Kaplan–Meier curves were used to evaluate the prognostic significance, and ROC curves were generated to assess the diagnostic efficiency [31]. Univariate and multivariate Cox Proportional Regression Hazard Models were prosecuted to assess the effects of diverse variables on overall survival and disease free survival. Subsequently, we downloaded the h.all.v7.0.symbols.gmt file as the reference target sets and performed gene set enrichment analysis (GSEA) to identify the important signaling pathways that PKMYT1 may be involved in ccRCC [32]. Gene sets with normalized *P*-value <0.05 and FDR <0.25 were considered as cutoff values.

To obtained genes highly correlated with the expression of PKMYT1, weighted gene co-expression network analysis (WGCNA) model was adopted to evaluate closely connected modules [33]. We first selected genes according to coefficient of variation values greater than 0.1. Then, we constructed a scale-free co-expression network for selected genes using the “WGCNA” R package [34]. All genes in the module in which PKMYT1 was located were selected. GO (Gene ontology) and KEGG (Kyoto Encyclopedia of Genes and Genomes) enrichment analyses were performed to visualize and annotate these genes highly associated with PKMYT1. Only terms with FDR-value of <0.05 were regarded markedly enriched.

### Statistical analysis

The RNA levels of wee family members were analyzed with paired student’s *t*-test. Prognostic values of PKMYT1 was evaluated by univariate and multivariate Cox regression analyses in ccRCC. The Kaplan–Meier (KM) curve combined with log-rank test were evaluated survival rate difference and expression level of PKMYT1. Pearson’s χ^2^ test was performed to analyze the relevance between PKMYT1 mRNA level and clinical data in ccRCC patients. Other statistical analysis and pictures drafting were performed with some packages (WGCNA, ggplot2, survival, pheatmap, rms and pROC) by R (version 4.0.3). For all statistical tests, *P*-value <0.05 was considered as considerable discrepancy.

## Supplementary Materials

Supplementary Figure 1

Supplementary Table 1

## References

[1] Siegel RL, Miller KD, Jemal A. Cancer statistics, 2019. CA Cancer J Clin. 2019; 69:7–34. 10.3322/caac.21551 30620402

[2] Ljungberg B, Bensalah K, Canfield S, Dabestani S, Hofmann F, Hora M, Kuczyk MA, Lam T, Marconi L, Merseburger AS, Mulders P, Powles T, Staehler M, et al. EAU guidelines on renal cell carcinoma: 2014 update. Eur Urol. 2015; 67:913–24. 10.1016/j.eururo.2015.01.005 25616710

[3] Siegel RL, Miller KD, Jemal A. Cancer statistics, 2020. CA Cancer J Clin. 2020; 70:7–30. 10.3322/caac.21590 31912902

[4] Siegel RL, Miller KD, Jemal A. Cancer statistics, 2015. CA Cancer J Clin. 2015; 65:5–29. 10.3322/caac.21254 25559415

[5] Xu J, Latif S, Wei S. Metastatic renal cell carcinoma presenting as gastric polyps: A case report and review of the literature. Int J Surg Case Rep. 2012; 3:601–4. 10.1016/j.ijscr.2012.08.009 22989776 PMC3484839

[6] Passer BJ, Nancy-Portebois V, Amzallag N, Prieur S, Cans C, Roborel de Climens A, Fiucci G, Bouvard V, Tuynder M, Susini L, Morchoisne S, Crible V, Lespagnol A, et al. The p53-inducible TSAP6 gene product regulates apoptosis and the cell cycle and interacts with Nix and the Myt1 kinase. Proc Natl Acad Sci U S A. 2003; 100:2284–9. 10.1073/pnas.0530298100 12606722 PMC151332

[7] Schmidt M, Rohe A, Platzer C, Najjar A, Erdmann F, Sippl W. Regulation of G2/M Transition by Inhibition of WEE1 and PKMYT1 Kinases. Molecules. 2017; 22:2045. 10.3390/molecules22122045 29168755 PMC6149964

[8] Wells NJ, Watanabe N, Tokusumi T, Jiang W, Verdecia MA, Hunter T. The C-terminal domain of the Cdc2 inhibitory kinase Myt1 interacts with Cdc2 complexes and is required for inhibition of G(2)/M progression. J Cell Sci. 1999; 112:3361–71. 10504341 10.1242/jcs.112.19.3361

[9] Lescarbeau RS, Lei L, Bakken KK, Sims PA, Sarkaria JN, Canoll P, White FM. Quantitative Phosphoproteomics Reveals Wee1 Kinase as a Therapeutic Target in a Model of Proneural Glioblastoma. Mol Cancer Ther. 2016; 15:1332–43. 10.1158/1535-7163.MCT-15-0692 27196784 PMC4893926

[10] Suganuma M, Kawabe T, Hori H, Funabiki T, Okamoto T. Sensitization of cancer cells to DNA damage-induced cell death by specific cell cycle G2 checkpoint abrogation. Cancer Res. 1999; 59:5887–91. 10606229

[11] Russell P, Nurse P. Negative regulation of mitosis by wee1+, a gene encoding a protein kinase homolog. Cell. 1987; 49:559–67. 10.1016/0092-8674(87)90458-2 3032459

[12] Carrassa L, Damia G. DNA damage response inhibitors: Mechanisms and potential applications in cancer therapy. Cancer Treat Rev. 2017; 60:139–51. 10.1016/j.ctrv.2017.08.013 28961555

[13] Sen T, Tong P, Diao L, Li L, Fan Y, Hoff J, Heymach JV, Wang J, Byers LA. Targeting AXL and mTOR Pathway Overcomes Primary and Acquired Resistance to WEE1 Inhibition in Small-Cell Lung Cancer. Clin Cancer Res. 2017; 23:6239–53. 10.1158/1078-0432.CCR-17-1284 28698200 PMC5882197

[14] Jin J, Fang H, Yang F, Ji W, Guan N, Sun Z, Shi Y, Zhou G, Guan X. Combined Inhibition of ATR and WEE1 as a Novel Therapeutic Strategy in Triple-Negative Breast Cancer. Neoplasia. 2018; 20:478–88. 10.1016/j.neo.2018.03.003 29605721 PMC5915994

[15] Znaor A, Lortet-Tieulent J, Laversanne M, Jemal A, Bray F. International variations and trends in renal cell carcinoma incidence and mortality. Eur Urol. 2015; 67:519–30. 10.1016/j.eururo.2014.10.002 25449206

[16] Yang K, Xiao Y, Xu T, Yu W, Ruan Y, Luo P, Cheng F. Integrative analysis reveals CRHBP inhibits renal cell carcinoma progression by regulating inflammation and apoptosis. Cancer Gene Ther. 2020; 27:607–18. 10.1038/s41417-019-0138-2 31570754 PMC7445881

[17] Zhang Q, Zhao X, Zhang C, Wang W, Li F, Liu D, Wu K, Zhu D, Liu S, Shen C, Yuan X, Zhang K, Yang Y, et al. Overexpressed PKMYT1 promotes tumor progression and associates with poor survival in esophageal squamous cell carcinoma. Cancer Manag Res. 2019; 11:7813–24. 10.2147/CMAR.S214243 31695486 PMC6707438

[18] Wang J, Wang L, Chen S, Peng H, Xiao L, E Du, Liu Y, Lin D, Wang Y, Xu Y, Yang K. PKMYT1 is associated with prostate cancer malignancy and may serve as a therapeutic target. Gene. 2020; 744:144608. 10.1016/j.gene.2020.144608 32234541

[19] Liu Y, Qi J, Dou Z, Hu J, Lu L, Dai H, Wang H, Yang W. Systematic expression analysis of WEE family kinases reveals the importance of PKMYT1 in breast carcinogenesis. Cell Prolif. 2020; 53:e12741. 10.1111/cpr.12741 31837068 PMC7046476

[20] Liu L, Wu J, Wang S, Luo X, Du Y, Huang D, Gu D, Zhang F. PKMYT1 promoted the growth and motility of hepatocellular carcinoma cells by activating beta-catenin/TCF signaling. Exp Cell Res. 2017; 358:209–16. 10.1016/j.yexcr.2017.06.014 28648520

[21] Jeong D, Kim H, Kim D, Ban S, Oh S, Ji S, Kang D, Lee H, Ahn TS, Kim HJ, Bae SB, Lee MS, Kim CJ, et al. Protein kinase, membrane-associated tyrosine/threonine 1 is associated with the progression of colorectal cancer. Oncol Rep. 2018; 39:2829–36. 10.3892/or.2018.6371 29658598

[22] Nakanishi M, Ando H, Watanabe N, Kitamura K, Ito K, Okayama H, Miyamoto T, Agui T, Sasaki M. Identification and characterization of human Wee1B, a new member of the Wee1 family of Cdk-inhibitory kinases. Genes Cells. 2000; 5:839–47. 10.1046/j.1365-2443.2000.00367.x 11029659

[23] Long HP, Liu JQ, Yu YY, Qiao Q, Li G. PKMYT1 as a Potential Target to Improve the Radiosensitivity of Lung Adenocarcinoma. Front Genet. 2020; 11:376. 10.3389/fgene.2020.00376 32411179 PMC7201004

[24] Sun QS, Luo M, Zhao HM, Sun H. Overexpression of PKMYT1 indicates the poor prognosis and enhances proliferation and tumorigenesis in non-small cell lung cancer via activation of Notch signal pathway. Eur Rev Med Pharmacol Sci. 2019; 23:4210–9. 10.26355/eurrev_201905_17925 31173292

[25] Utz PJ, Anderson P. Life and death decisions: regulation of apoptosis by proteolysis of signaling molecules. Cell Death Differ. 2000; 7:589–602. 10.1038/sj.cdd.4400696 10889504

[26] Zhang QY, Chen XQ, Liu XC, Wu DM. PKMYT1 Promotes Gastric Cancer Cell Proliferation and Apoptosis Resistance. Onco Targets Ther. 2020; 13:7747–57. 10.2147/OTT.S255746 32801781 PMC7414979

[27] Chaffer CL, San Juan BP, Lim E, Weinberg RA. EMT, cell plasticity and metastasis. Cancer Metastasis Rev. 2016; 35:645–54. 10.1007/s10555-016-9648-7 27878502

[28] Beerling E, Seinstra D, de Wit E, Kester L, van der Velden D, Maynard C, Schäfer R, van Diest P, Voest E, van Oudenaarden A, Vrisekoop N, van Rheenen J. Plasticity between Epithelial and Mesenchymal States Unlinks EMT from Metastasis-Enhancing Stem Cell Capacity. Cell Rep. 2016; 14:2281–8. 10.1016/j.celrep.2016.02.034 26947068 PMC4802222

[29] Wang XM, Li QY, Ren LL, Liu YM, Wang TS, Mu TC, Fu S, Liu C, Xiao JY. Effects of MCRS1 on proliferation, migration, invasion, and epithelial mesenchymal transition of gastric cancer cells by interacting with Pkmyt1 protein kinase. Cell Signal. 2019; 59:171–81. 10.1016/j.cellsig.2019.04.002 30953699

[30] Fujiwara T, Hirooka S, Ohbayashi R, Onuma R, Miyagishima SY. Relationship between Cell Cycle and Diel Transcriptomic Changes in Metabolism in a Unicellular Red Alga. Plant Physiol. 2020; 183:1484–501. 10.1104/pp.20.00469 32518202 PMC7401142

[31] Sing T, Sander O, Beerenwinkel N, Lengauer T. ROCR: visualizing classifier performance in R. Bioinformatics. 2005; 21:3940–1. 10.1093/bioinformatics/bti623 16096348

[32] Subramanian A, Tamayo P, Mootha VK, Mukherjee S, Ebert BL, Gillette MA, Paulovich A, Pomeroy SL, Golub TR, Lander ES, Mesirov JP. Gene set enrichment analysis: a knowledge-based approach for interpreting genome-wide expression profiles. Proc Natl Acad Sci U S A. 2005; 102:15545–50. 10.1073/pnas.0506580102 16199517 PMC1239896

[33] Horvath S, Dong J. Geometric interpretation of gene coexpression network analysis. PLoS Comput Biol. 2008; 4:e1000117. 10.1371/journal.pcbi.1000117 18704157 PMC2446438

[34] Langfelder P, Horvath S. WGCNA: an R package for weighted correlation network analysis. BMC Bioinformatics. 2008; 9:559. 10.1186/1471-2105-9-559 19114008 PMC2631488

