# Correction for: PKMYT1, exacerbating the progression of clear cell renal cell carcinoma, is implied as a biomarker for the diagnosis and prognosis

**DOI:** 10.18632/aging.206113

**Published:** 2024-09-15

**Authors:** Juan Chen, Xiaoliang Hua, Heying Chen, Xiangmin Qiu, Haibing Xiao, Shengdong Ge, Chaozhao Liang, Qin Zhou

**Affiliations:** 1 The Ministry of Education Key Laboratory of Laboratory Medical Diagnostics, The College of Laboratory Medicine, Chongqing Medical University, Chongqing 400016, China; 2 Department of Urology, The First Affiliated Hospital of Anhui Medical University, Hefei, China; 3 Anhui Province Key Laboratory of Genitourinary Diseases, Anhui Medical University, Hefei, China; 4 The Institute of Urology, Anhui Medical University, Hefei, China

**Keywords:** renal cell carcinoma, PKMYT1, EMT, prognosis, diagnosis

**This article has been corrected:** The authors forgot to add one more source of funding from Scientific and Technological Research Program of Chongqing Municipal Education Commission Grant No. KJQN202100418. The corrected text is presented below.

