# Supplementary material for: PKMYT1, exacerbating the progression of clear cell renal cell carcinoma, is implied as a biomarker for the diagnosis and prognosis
Source: Aging (Albany NY). 2021 Dec 27;13(24):25778–98. doi: 10.18632/aging.203759 (PMC8751600; doi:10.18632/aging.203759)
Supplement: Supplementary Figure 1 [file aging-13-203759-s001.pdf]

SUPPLEMENTARY FIGURE

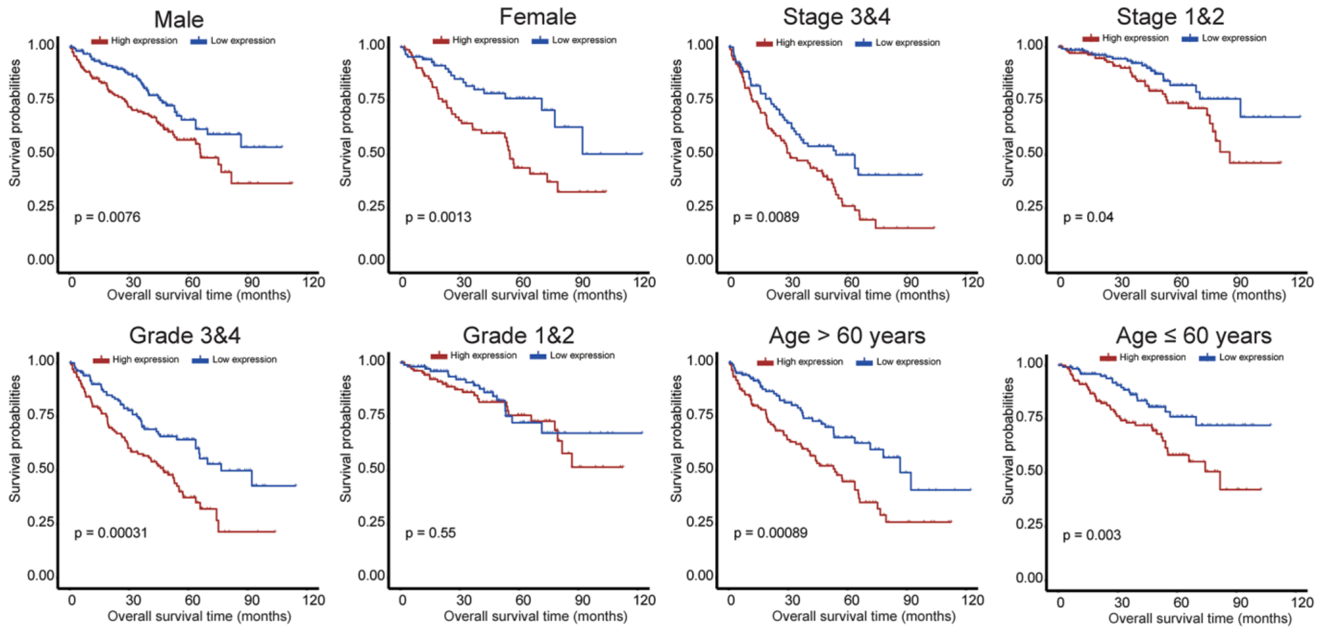

Supplementary Figure 1. Survival analysis for ccRCC patients in different subgroups. The PKMYT1 high expression group of tended to have worse prognosis in males, females, stage 3&4, stage 1&2, grade 3&4, age >60 years, and age ≤60 years. Survival analysis exhibited no significant difference in the grade 1&2 subgroups.
